# Tropism of Avian Influenza A (H5N1) Virus to Mesenchymal Stem Cells and CD34^+^ Hematopoietic Stem Cells

**DOI:** 10.1371/journal.pone.0081805

**Published:** 2013-12-10

**Authors:** Maytawan Thanunchai, Pumaree Kanrai, Suwimon Wiboon-ut, Pilaipan Puthavathana, Suradej Hongeng, Arunee Thitithanyanont

**Affiliations:** 1 Department of Microbiology, Faculty of Science, Mahidol University, Bangkok, Thailand; 2 Department of Microbiology, Faculty of Medicine Siriraj Hospital, Mahidol University, Bangkok, Thailand; 3 Department of Pediatrics, Faculty of Medicine, Ramathibodi hospital, Mahidol University, Bangkok, Thailand; Boston University School of Medicine, United States of America

## Abstract

The presence of abnormal hematologic findings such as lymphopenia, thrombocytopenia, and pancytopenia were diagnosed in severe cases of avian influenza A H5N1. Whether direct viral dissemination to bone marrow (BM) cells causes this phenomenon remains elusive. We explore the susceptibility of the two stem cell types; hematopoietic stem cells (HSCs) and mesenchymal stromal cells (MSCs) isolated from human BM cells or cord blood, to infection with avian H5N1 viruses. For the first time, we demonstrated that the H5N1 virus could productively infect and induce cell death in both human stem cell types. In contrast, these activities were not observed upon human influenza virus infection. We also determined whether infection affects the immunomodulatory function of MSCs. We noted a consequent dysregulation of MSC-mediated immune modulation as observed by high cytokine and chemokine production in H5N1 infected MSCs and monocytes cocultures. These findings provide a better understanding of H5N1 pathogenesis in terms of broad tissue tropism and systemic spread.

## Introduction

The highly pathogenic avian influenza A virus of the H5N1 subtype was originally endemic to poultry, but crossed the avian-human species barrier. It has emerged as a highly fatal infectious disease in the human population with a 60% mortality observed in more than ten countries, as reported to the World Health Organization since 2003 [1], [2]. The pathologic process of H5N1 patients is initial presentation with fever and respiratory symptoms including cough and shortness of breath [3]. In addition, severe cases are characterized by fulminant viral pneumonia, acute respiratory distress syndrome, multi-organ failure and death [4]. Upon infection, the virus can spread from the lungs to other organs [5] and can also pass to the fetus [4], [5], causing systemic disease which leads to an unusually high mortality rate. The fatal outcome of H5N1 viral infection has been linked to the presence of high viral load, hypercytokinemia and the associated reactive hemophagocytosis syndrome [4]. Hematologic abnormalities were commonly observed in severe cases, including lymphoid depletion, leucopenia, thrombocytopenia and pancytopenia, which are likely related to bone marrow (BM) suppression, and/or virus-associated hemophagocytosis. Despite the known systemic spread of the virus, there have been no reports of viral isolation from BM itself, even when the viral antigen was detected in other samples from the same autopsy subject [6]. If the virus-mediated BM suppression is a possible factor contributing to the observed substantial cell loss, hematologic abnormalities, and hyperinflammatory cytokine production, it is thus intriguing to determine whether the observed suppression is a result of direct invasion of virus.

Bone marrow (BM) is an important source from which progenitor cells are generated. It contains two types of progenitor cells; hematopoietic stem cells and non-hematopoietic stem cells which can differentiate into blood cells and cells of mesenchymal lineages (osteoblasts, chondrocytes, adepocytes, etc.), respectively [7]. Hematopoietic stem cells (HSCs) have renewal and differentiation abilities [8]. HSCs are able to migrate out of the bone marrow into the blood circulation system [9]. CD34^+^ cell is a population that includes hematopoietic stem cells (HSCs), myeloid, erythroid and lymphoid progenitors [10]. Recently, umbilical cord and placenta were recognized as rich sources of HSCs for isolation [11] which share similar characteristics as HSCs of BM [12]. MSCs have many of the key biological characteristics that make up the defining criteria for accepted stem cells as reviewed above. In BM, MSCs act as supporting cells that regulate the normal hematopoiesis process including growth, maturation, differentiation and survival of HSCs [13]. Due to its ability to differentiate, MSCs can regenerate damaged tissues by differentiating into the particular phenotype of the damaged cells [14]. MSCs also exert immunomodulatory activities by suppressing NK, T, B and monocyte-derived dendritic cells (MoDCs) proliferation and function [15]–[17]. These properties appear to be more important for therapeutics designed to regulate the immune response under conditions such as tissue injury, transplantation, and autoimmunity [18].

The fact that BM is a rich source of progenitor cells [19] and a possible target of H5N1-induced hematologic abnormalities [20], led us to investigate the direct infection of H5N1 virus in BM. In this study, we demonstrated that highly pathogenic avian influenza (HPAI) H5N1 virus could productively infect and replicate in CD34^+^ HSCs and MSCs. Sialic acid (SA) receptors on the target cells surface were believed to be the major receptor mediating virus binding and entry. H5N1 viral infection induced cell death in both CD34^+^ HSCs and MSCs. We also observed that pro-inflammatory cytokines and chemokines were enhanced in cocultures of MSCs and primary monocytes indicating that H5N1 infection altered the immunomodulative effect of MSCs.

## Materials and Methods

### Umbilical Cord Blood (CB) and BM Samples

Umbilical cord blood (CB) was obtained from full term newborns and collected in sterile collection bags containing anti-coagulant citrate-phosphate dextrose. Bone marrow (BM) samples were aspirated from the posterior superior iliac spine of healthy donors. The CD34^+^ expressing hematopoietic stem cells (CD34^+^ HSCs) were isolated from the above two sources, whereas, Mesenchymal Stromal Cells (MSCs) were BM derived. Stem cells were isolated no later than 15 hours (h) after collection of the material. All samples for the present study were obtained with written informed consent from all the donors as per the approval of Institutional Review Board, Faculty of Medicine Ramathibodi Hospital, Mahidol University, Thailand.

### Isolation and culture of CD34^+^ HSCs and CD14^+^ Monocytes

Mononuclear Cells (MCs) were isolated from both CB (CBMCs) and BM (BMMCs) using IsoPrep (Robbins Scientific, Canada) density centrifugation. CD34^+^ HSCs were isolated from both CBMCs and BMMCs using CD34 MACS Microbeads (Miltenyi, Gladbach, Germany) following the manufacturer's instructions. The efficiency of CD34^+^ HSCs purification was determined by staining of isolated cell surface markers with phycoerythrin (PE)-conjugated anti-CD34 monoclonal antibody (Miltenyi, Gladbach, Germany) for 30 min at 4°C. Cells were washed in PBS and analyzed by Flow Cytometry (Becton-Dickinson Pharmingen, Heidelberg, Germany) on an instrument equipped with an argon laser tuned at 488 nm. The percentage of obtained CD34^+^ HSCs ranged from 98% to 99% in all of the purified CB and BM samples. Cells expressing >98% CD34 antigen were cultured in Stemline II media (Sigma, USA) supplemented with specific hematopoietic growth factor cocktail that included 50 ng/ml stem cell factor, 50 ng/L rh-IL-6 and 50 ng/L rh-IL-3 (R&D Systems). CD14^+^ monocytes used in the cocultures study were isolated as previously described [21]. Briefly, peripheral blood mononuclear cells (PBMCs) were obtained by centrifugation using IsoPrep, and CD14^+^ monocytes were isolated by using CD14 MACs Microbeads. Monocytes were cultured in RPMI 1640 media (Gibco, Life Technology, Rockville, Md., USA) supplemented with 10% fetal bovine serum (FBS), 2 mM L-glutamine and 1% penicillin-streptomycin (Pen/Strep).

### MSC purification and culture

BMMCs were separated by density gradient centrifugation with IsoPrep. Briefly, 10 ml of heparinized bone marrow samples were mixed in with an equal volume of Dulbecco's Modified Eagle's Medium (DMEM) (Gibco, Life Technology, Rockville, Md., USA) and centrifiuged at 1000 g for 30 min at room temperature. The interface mononuclear cells were collected and washed twice with DMEM. Total cell count and viablility were evaluated by 0.2% Trypan blue. A total of 2×10^6^ cells/ml of BMMCs were cultured in DMEM complete medium supplemented with 10% FBS and 1% Pen/Strep at 37°C, 5% CO_2_. After 72 hours of cultivation, non-adherent cells were discarded and this process was repeated every four days. Upon reaching 90% confluency, MSCs were trypsinized by 0.05% trypsin-EDTA and passaged for the next expansion.

### Lectin staining

To identify sialyoligosaccharides reactive with SAα2,3Gal- or SAα2,6Gal-specific lectins, 1×10^5^ each of HSCs and MSCs were incubated with fluorescein isothiocyanate (FITC)-labelled lectins Sambucus nigra agglutinin (SNA) and Peridinin Chlorophyll Protein Complex (PerCP)-labelled Maackia amurensis agglutinin (MAA) (Boehringer Mannheim Biochemicals) which primarily detects SAα2,6galactose and SAα2,3galactose, respectively [22]. After three washes in ice cold PBS, samples were analyzed by flow cytometry (Becton Dickinson).

### Influenza virus infection of HSCs and MSCs

The different subtypes of influenza A virus: HPAI H5N1 (A/open-billed stork/Nakhonsawan/BBD0104F/04) and human influenza A viruses; H1N1 (A/WS/33) and H3N2 (A/Hong Kong/8/68) were propagated and quantified as previously described [21]. These strains were used throughout this study. For CD34^+^ HSCs, cultured cells were washed with serum free media and allowed to adsorb virus for 1 hour for each type of viruses at a multiplicity of infection (MOI) of 1 and 10. All experiments with H5N1 virus were performed inside a bio-safety level 3 facility by trained researchers. Following adsorption, cells were again washed with serum free medium and cultured in Stemline II media supplemented with specific hematopoietic growth factor cocktails. For H1N1 and H3N2 virus infection, the same media was used but contained 0.02 ug/ml of TPCK trypsin (Sigma-Aldrich) was added into the wells [23]. Simultaneously, MSCs were plated one day prior to infection. After the cultures attained 100% confluent growth, cells were washed with serum free media and adsorbed for 1 hour with virus MOI 1 and 10. Following adsorption, cells were washed three times with serum free growth media and cultured in serum-free media. As a control, H5N1 virus was heat-killed at 56°C for 30 min [24].

### Generation of reverse genetics virus

A fragment of the HA gene covering the multibasic cleavage site had been modified to a low-pathogenic sequence was cloned into pHw2000 plasmid, sequenced, and used for construction of reverse genetic (rg) viruses as described previously [25]. Briefly, HEK-293 cells cocultured with MDCK cells on a 6-well plate were transfected with wild-type HA or mutant HA together with the other seven genomic segments of A/Puerto Rico/8/34 (H1N1) to generate the rgPR8-H5 and rgPR8-H5monobasic viruses, respectively.

### Cocultures of MSCs and CD14^+^ monocytes

MSCs were seeded the day before the experiment in 24-well flat-bottom plates in RPMI 1640 supplemented with 10% FBS and 1% Pen/Strep at 37°C, 5% CO_2_ overnight. Freshly isolated 3×10^5^ CD14^+^ monocytes were added to each well (MSC:monocyte ratio 1∶5). Controls included monocultured MSCs and monocytes.

Confluent monolayers of cocultures, MSCs and CD14^+^ monocytes were washed with serum free media and then, infected with H5N1 virus at an MOI of 0.04, and incubated at 37°C for 1 hour. Untreated cells were used as a control. Following infection, the inoculum was removed, cells were washed with serum free media and then 1 ml of RPMI 1640 media supplemented with 10% FBS was placed into the wells. Cytokines produced from supernatants of infected cocultures, MSCs monoculture and monocytes monoculture were determined at 24 hours post infection by a Bio-plex Pro Human Cytokine Assay.

### Immunofluorescence assay for detection of influenza A viral antigen

An immunofluorescence assay was performed on 2.5×10^4^ cells grown on a slide culture. At 24 hours post infection (p.i.), cells were fixed with 4% paraformaldehyde in PBS and permeablised by cytofix/cytoperm solution (BD Biosciences). Viral nucleoprotein (NP) was stained with FITC-conjugated antibody (green) (DAKO). CD34^+^ cells were counterstained with phycoerythrin (PE)-conjugated anti-CD34 monoclonal antibody (red) (Miltenyi Biotec). The stained cells were analyzed using a lasers canning confocal microscope (LSM510 META; Carl Zeiss) at the Division of Medical Molecular Biology, Office for Research and Development, Faculty of Medicine Siriraj Hospital, Mahidol University.

### Flow cytometry for determination of viral infection and cell surface markers

The surface markers of cells were analyzed to detect CD34^+^ and CD14^+^ cells using PE and PerCP-conjugated specific monoclonal antibodies, respectively, according to the manufacturer's instructions (MACS, Germany and BD, USA). For detection of viral antigen, all infected cells were incubated with FITC-conjugated NP antibody (green) (Chemicon, Temecula, CA.). These cells were washed, resuspended in 3.7% formaldehyde prior to analysis by flow cytometry.

### Real-time PCR

Infected MSCs were harvested at various time points, total viral RNA was extracted by use of an RNeasy minikit (Qiagen) following the manufacturer's instructions. Cells were washed for three times with PBS to remove free virions. cDNA was synthesized from viral genomic RNA using oligodT primers and AMV reverse transcriptase (Promega). These cDNA samples were used as template. The M gene of the H5N1 virus was amplified as previously described [21] using HotStarTaq DNA Polymerase (Qiagen). Gene copies were quantified on the basis of a SYBR green fluorescence signal by Rotor-Gene 3000 (Corbett Robotics). The standard curve was generated using a serial dilution of plasmid (from 1-10^7^ copies) containing the respective cloned gene target. The results were standardized due to the variable quantities of RNA and cDNA and finally expressed as numbers of target genes.

### Plaque assay

Culture supernatants were collected from infected CD34^+^ HSCs and MSCs at various time points. The viral titers in supernatants were quantified by plaque assay. In brief, confluent monolayers of MDCK cells were inoculated with 10-fold dilutions of H5N1 virus and incubated at 37°C for 1 h. The inoculum was removed, and cells were washed and overlaid with plaque medium containing 2% agarose. After two days, cells were stained with 1.25% crystal violet, and the plaque numbers were evaluated.

### Apoptosis detection

Following viral infection of 1×10^5^ cells/well on a 24-well plate at an MOI of 1 or 10, CD34^+^ HSCs and MSCs were cultured in a specific media as previously described for 16 h. Parallel samples were treated with DNaseI as a positive control. Apoptotic cells were characterized by positive terminal deoxynucleotidyltransferase-mediated dUTP-biotin nick end labeling (TUNEL) staining according to the protocol provided by the manufacturer. (In Situ Cell Death Detection Kit, TMR red, Roche). Briefly, 16 h after infection with H5N1, H1N1, or H3N2, cells were fixed with 4% paraformaldehyde and permeabilised by cytofix/cytoperm solution (BD Biosciences). After that, cells were then incubated in the labeling reaction mixture containing terminal deoxynucleotidyl transferase enzyme, TMR red-conjugated nucleotide at 37°C for 1 hour and cells were washed with PBS several times. To detect viral antigen after TUNEL staining, cells were incubated at 4°C for 30 min with FITC-conjugated anti-NP antibody as mentioned earlier. Both CD34^+^ HSCs and MSCs were counterstained with DAPI, and then imaged with a laser scanning confocal microscope (LSM510 META; Carl Zeiss).

### Measurement of cytokines

Cytokine protein levels were measured (at 24 hours p.i.) by a Bio-Plex Pro Human Cytokine Assay (Bio-Rad, Hercules, CA) following the manufacturers' instructions. Cytokine levels were measured in triplicate and compared against a standard curve.

### Statistical analysis

The Statistical analysis was performed on SPSS Statistics 17.0 using one way analysis of variance (ANOVA) to compare susceptibilities of stem cells to H5N1 infection and to H1N1 or H3N2 infections with statistical significance set at *p*<0.05

## Results

### BM cells express receptors for influenza virus

**Figure 1:**
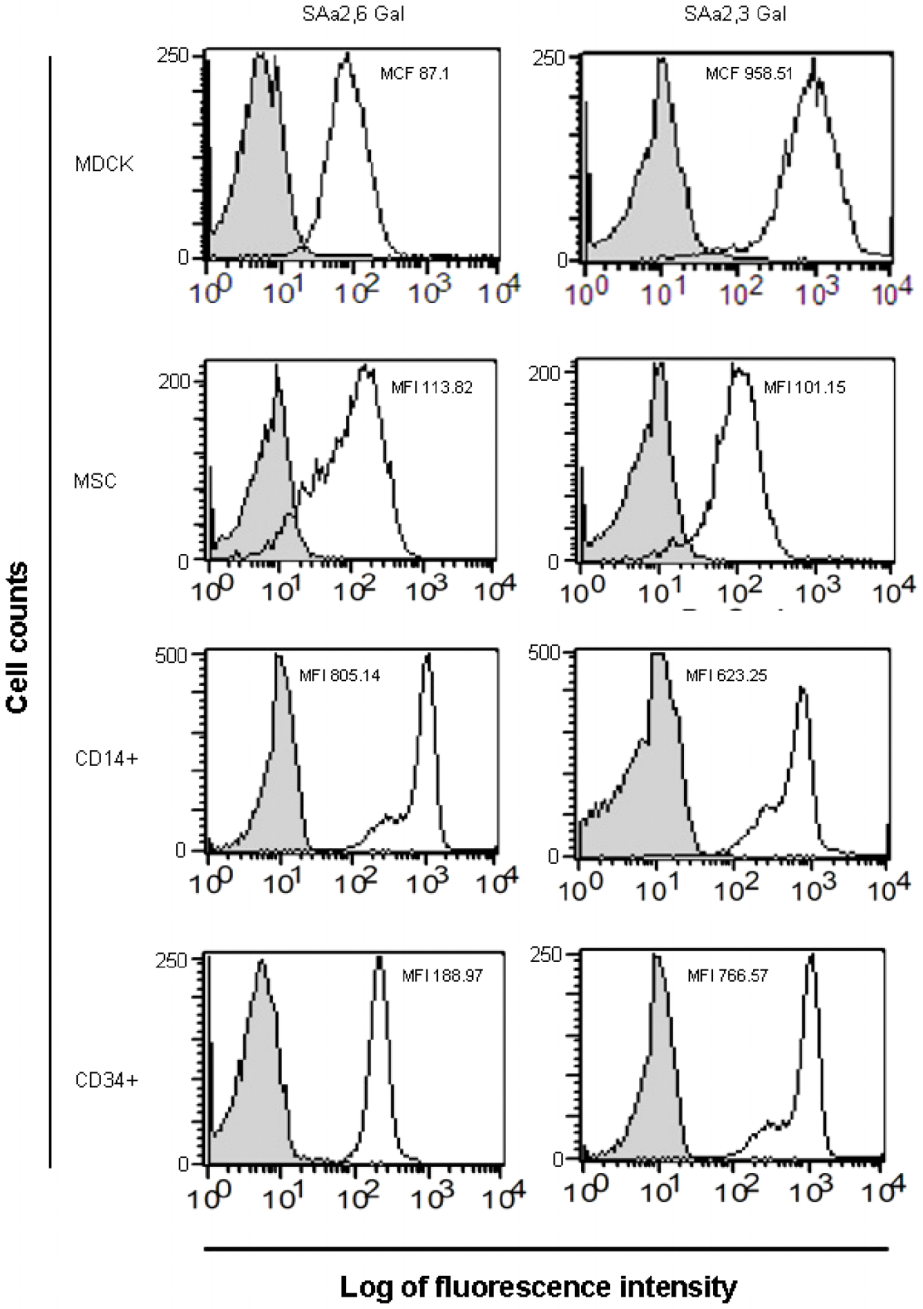
Relative amounts of Sialic Acid (SA) on the surface of various cell types. CD34^+^ HSCs, MSCs, CD14^+^ monocytes and MDCK cells were incubated with SNA and MAA which are specific for α2,3 and α2,6 SA, respectively, and determined by flow cytometry. The profile shown depicts cell number as a function of log fluorescence intensity of SAα2,3Gal and SAα2,6Gal specific lectin-reactive oligosaccharide on the cell surface. Relative mean fluorescent intensity (MFI) and mean channel fluorescence (MCF) which describe the brightness of fluorescein parameter were calculated by deducting MFI and MCF of negative control (Grey). Data are representative of three separate experiments.

Sialic acid (SA) on the cell surface is widely recognized as an influenza virus receptor, which can mediate influenza virus binding and fusion. SA linked to galactose (SAα2,3-Gal) was distributed in the avian gastrointestinal tract, and human lower respiratory tract, whereas, SAα2,6-Gal was expressed in the human upper respiratory tract [22], [26], [27]. However, there have been no reports on SA receptor expression on stem cells. Therefore, we investigated whether BM stem cells expressed SA receptors. We stained isolated CD34^+^ HSCs and MSCs with MAA and SNA specific for SAα2,3-Gal and SAα2,6-Gal, respectively. We demonstrated that CD34^+^ HSCs and MSCs expressed receptors of both avian (SAα2,3-Gal) and human (SAα2,6-Gal) influenza viruses (Figure 1). MDCK and CD14^+^ monocytes were used as a control as they are known targets of influenza virus infection and have been reported to express both SA receptors [21]. This result indicated that CD34^+^ HSCs and MSCs could be targets of avian and human influenza viruses.

### HSCs are susceptible to avian influenza (H5N1) virus but not to human influenza virus

**Figure 2:**
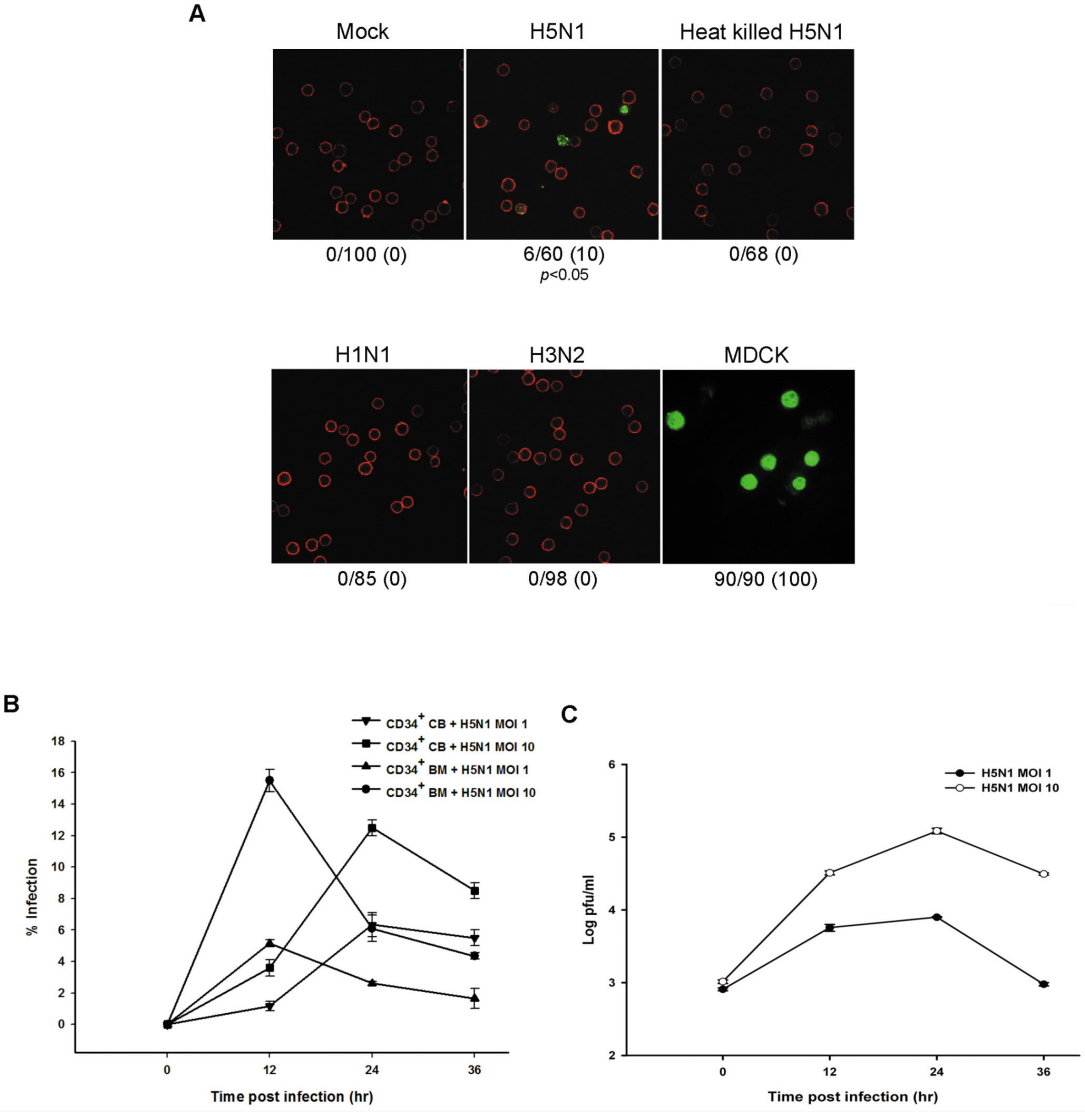
Infection and Replication of CD34^+^ HSCs by H5N1 virus. (**A**) CD34^+^ HSCs from CB were infected with both live and heat-killed avian (H5N1) and human influenza viruses (H1N1 and H3N2) at an MOI of 10 for 24 h. MDCK infected with H5N1 at MOI 1 for 12 hours was used as a positive control. Cells were fixed and permeabilized for intracellular staining of Influenza nucleoprotein (NP; green). CD34^+^ cells were counterstained with anti-human CD34-PE (red), and then examined by confocal microscopy and photographed at a ×200 magnification. The percentage of NP-positive (NP+) cells was obtained by counting the number of NP+ cells per total cells in five random fields. (**B**) CD34^+^ HSCs from both BM and CB sources were exposed to either a high (MOI 10) or low (MOI 1) dose of H5N1 virus. The expression of viral antigens was analyzed by flow cytometry. (**C**) Supernatants of infected CD34^+^ HSCs at MOI 1 and 10 were collected at different time points after infection and then titrated on MDCK cells to measure virus production by plaque assay. The results were obtained from three different experiments and are presented as means plus standard errors. *P* value<0.05 indicate the significance of H5N1 infection compared with human influenza viruses

To investigate whether H5N1 virus could infect hematopoietic stem cells, CD34^+^ HSCs from BM and CB were isolated, purified, and then infected with H5N1 virus as described in the materials and methods section. At 24 hours post-infection (p.i.), viral antigen (NP) was expressed in CD34^+^ HSCs when infected with live H5N1 virus but not heat-killed virus (Figure 2A). In contrast, CD34^+^ HSCs were completely resistant to human influenza viruses (H1N1 and H3N2) as shown in immunofluorescent staining in Figure 2A and flow cytometry in Figure S1. The percentage of NP-positive (NP+) cells of H5N1 infection was statistically different from human influenza viruses infection with *P* value <0.05 (Figure 2A). Using flow cytometry, we demonstrated that both CB and BM-derived CD34^+^ HSCs were susceptible to a high MOI of H5N1 virus, but showed less susceptibility at low MOI (Figure 2B). Similar susceptibility levels were observed among different H5N1 strains (Figure S1). We continued investigating whether these cells support the production of new infectious virus particles. Culture supernatants were collected from infected cells at various time points to determine the viral output using a standard plaque assay. The level of released infectious H5N1 virus particles from CD34^+^ HSCs was one-log increased from 0 to 12 h, and peaked at 24 h p.i. at high MOI, while increased by less than one log at 12 h p.i. and appeared to be stable afterward at low MOI (Figure 2C). These findings suggest that the H5N1 virus may have stem cell tropism that enables virus to infect and replicate in CD34^+^ HSCs.

### MSCs are highly susceptible to avian influenza H5N1 virus

**Figure 3:**
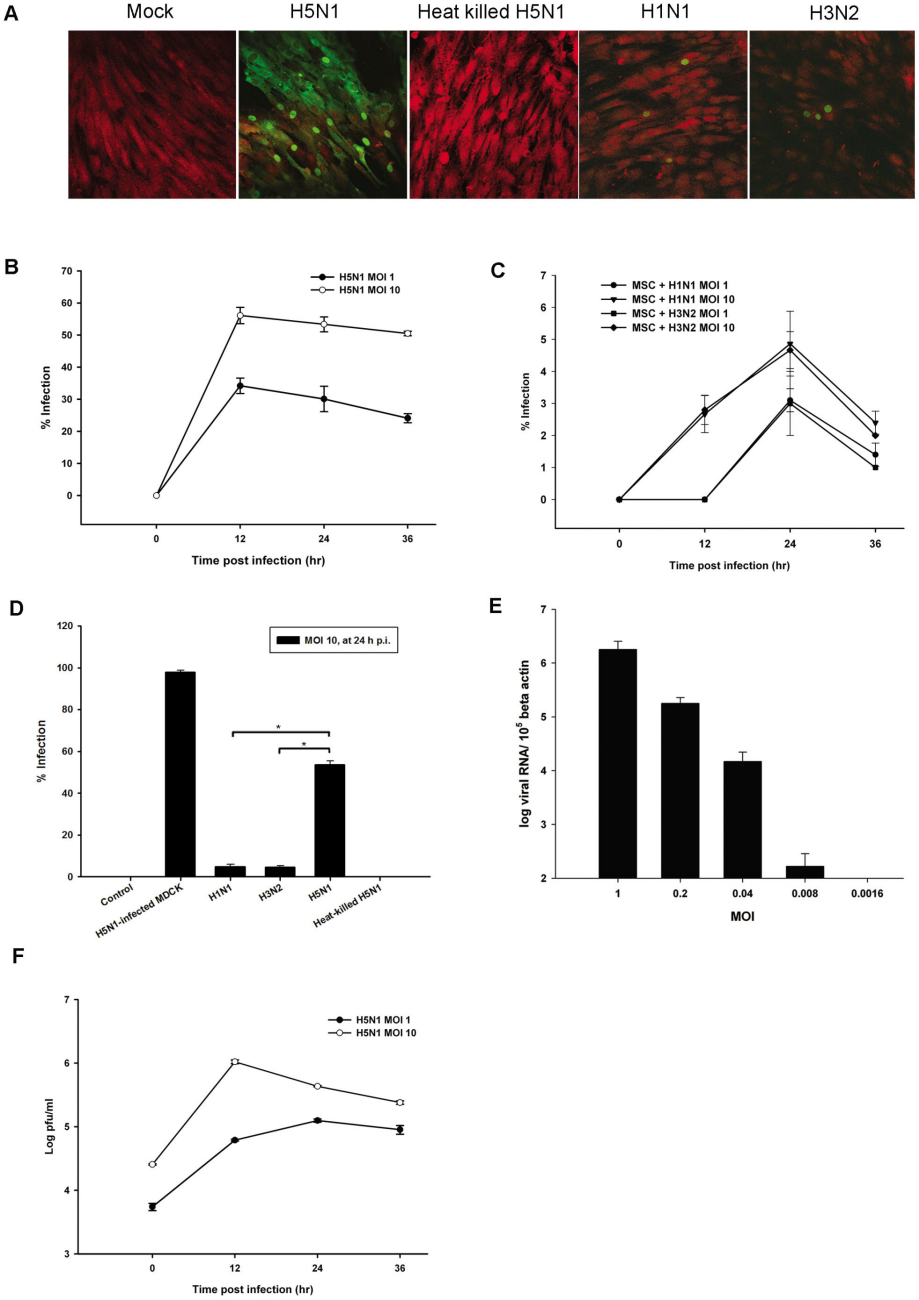
Avian influenza could infect and replicate in MSCs. (**A**) The ability of avian influenza H5N1 to infect MSCs was compared to human influenza viruses. Cells were infected with H5N1; live or heat-inactivated, H1N1 and H3N2 at an MOI of 10. After 24 h, cells were stained and examined by confocal microscopy for the expression of viral antigens (green) with Evan's blue (red) as a counterstain. The percentages of infected MSCs at high and low MOIs of (**B**) H5N1 virus and (**C**) human influenza viruses were determined by flow cytometry. (**D**) Percent infection of MSCs infected mock, H1N1, H3N2, H5N1, and heat-killed H5N1 at MOI 10 for 24 hours. MDCK infected with H5N1 at MOI 1 for 12 hours was used as a positive control. (**E**) Real-time PCR of H5N1 M gene in MSCs after infection with H5N1 at various doses for 12 h. The amount of the virus RNA was standardized by beta actin. (**F**) Production of H5N1 virus from infected MSCs at various time points was measured by plaque assay. The results represent the means and SD of three independent experiments. *P*<0.05 indicates statistically significant differences between H5N1 and human influenza viruses infections.

We next investigated the susceptibility of non-hematopoietic stem cells, MSCs to H5N1 virus. MSCs were isolated from BM. Once a homogeneous cell culture was obtained upon culturing the cells in an appropriate media, cells were tested for surface markers characteristics. Isolated MSCs expressed CD73, CD90, and CD105, but not CD34 and CD45 (data not shown). MSCs were infected with H5N1, H1N1 and H3N2 viruses. Surprisingly, MSCs were highly susceptible to H5N1 virus which live virus was required (Figure 3A). The susceptibility of MSCs to infection was shown in different levels correlated with the exposure dose (Figure 3B and 3E). MSCs showed a limited infection to human influenza virus strains as seen by both fluorescence microscopy and flow cytometry which percent infection was significantly different from H5N1 infection (Figure 3A, 3C, and 3D). Moreover, H5N1 virus had a high replicative efficiency in MSCs with viral production increasing by more than one-log from 0 to 12 h p.i at an MOI of 10 (Figure 3F). An MOI of 0.008 (one virus particle per every 120 target cells) was sufficient to infect MSCs to generate at least a two-log increase in new H5N1 virus particles at 36 h after infection (Figure S2). This phenomenon is restricted only to HPAI H5N1 since the percentages of infection of rgPR8-H5 which represents HPAI H5N1 was significantly higher than low-pathogenic rgPR8-H5monobasic in MSCs with *P* value <0.05 (Figure S3). These findings indicated that H5N1 virus could infect and replicate in MSCs efficiently in order to expand its host range in the BM environment.

### Avian influenza virus infection kills CD34^+^ HSCs and MSCs

**Figure 4:**
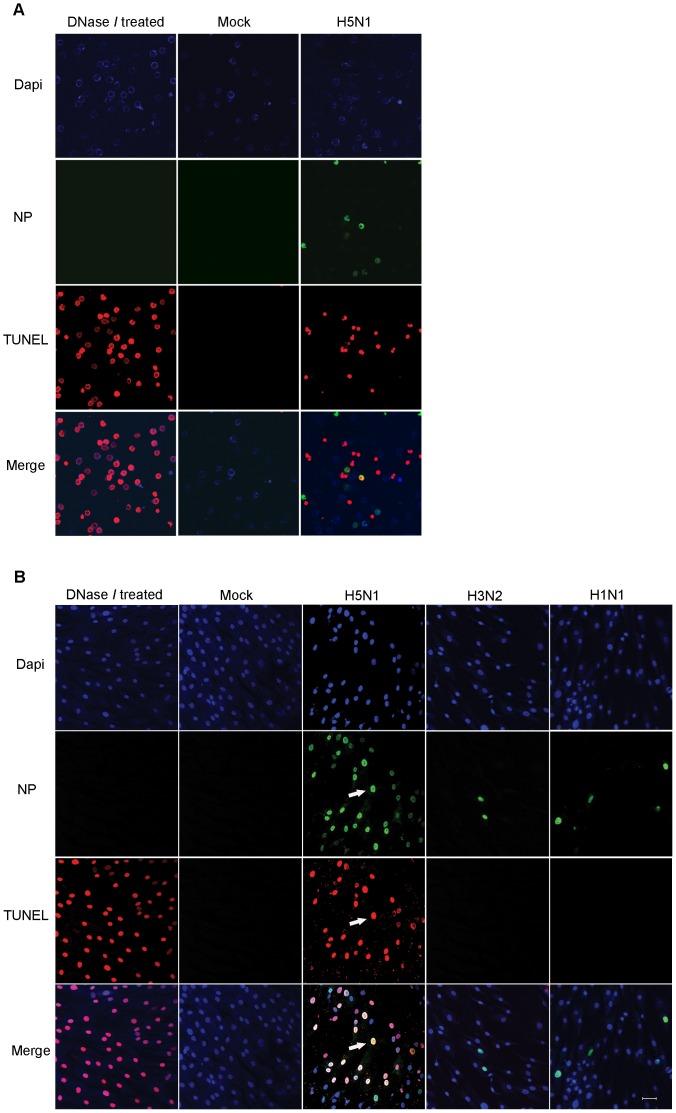
H5N1 infection induced marked CD34^+^ HSC and MSC apoptosis. (**A**) CD34^+^ HSCs were infected with H5N1 virus at an MOI of 10, whereas (**B**) MSCs were infected with either avian or human influenza virus at the same MOI as CD34^+^ HSCs. Parallel samples were treated with DnaseI which breaks double stranded DNA as a positive control. After 18 h, cells were fixed, permeabilized and multiple stained using Terminal deoxynucleotidyl transferase–mediated dUTP-biotin nick end-labeling (TUNEL) staining (red), FITC-labeled anti-NP (green) and DAPI (blue). Arrows in B. indicate cells that simultaneously expressed intracellular viral antigen and apoptotic signal. The samples were examined on a confocal microscopy and photographed at a ×200 magnification.

Once human stem cells were infected, the number of viable cells decreased (data not shown). We suspected that this was related to apoptosis of these cells. With this thought, we investigated the fate of MSCs and CD34^+^ HSCs after infection. Apoptotic cells were characterized by positive terminal deoxynucleotidyltransferase-mediated dUTP-biotin nick end labeling (TUNEL) staining (In Situ Cell Death Detection Kit, TMR red, Roche). TUNEL assay confirmed that live H5N1 virus could induce apoptosis in both CD34^+^ HSCs and MSCs, whereas, human influenza viruses and viral proteins of heat-killed H5N1 could not (Figure 4A and 4B). Virus-induced cell death was observed in most of H5N1-infected MSCs (Figure 4B). The percentages of merge signals (NP+TUNEL+) were comparable with single positive signal (NP+ or TUNEL+) in H5N1-infected MSCs as shown in Figure S4, which was correlated with co-localization of multiple colors in merge picture in Figure 4B. However, apoptosis was also observed in some of the uninfected CD34^+^ HSCs population as the number of TUNEL+; apoptosis without infection was significantly higher than NP+TUNEL+; infected and apoptosis (Figure S4). This data was correlated with non-localizing red signal in merge picture of H5N1-infected CD34^+^ HSCs in Figure 4A. This finding indicated that H5N1 virus caused apoptosis in stem cells by a direct effect of infection in MSCs, but by an indirect one on CD34^+^ HSCs.

### Avian influenza virus subverts MSCs-mediated immune modulation

**Figure 5:**
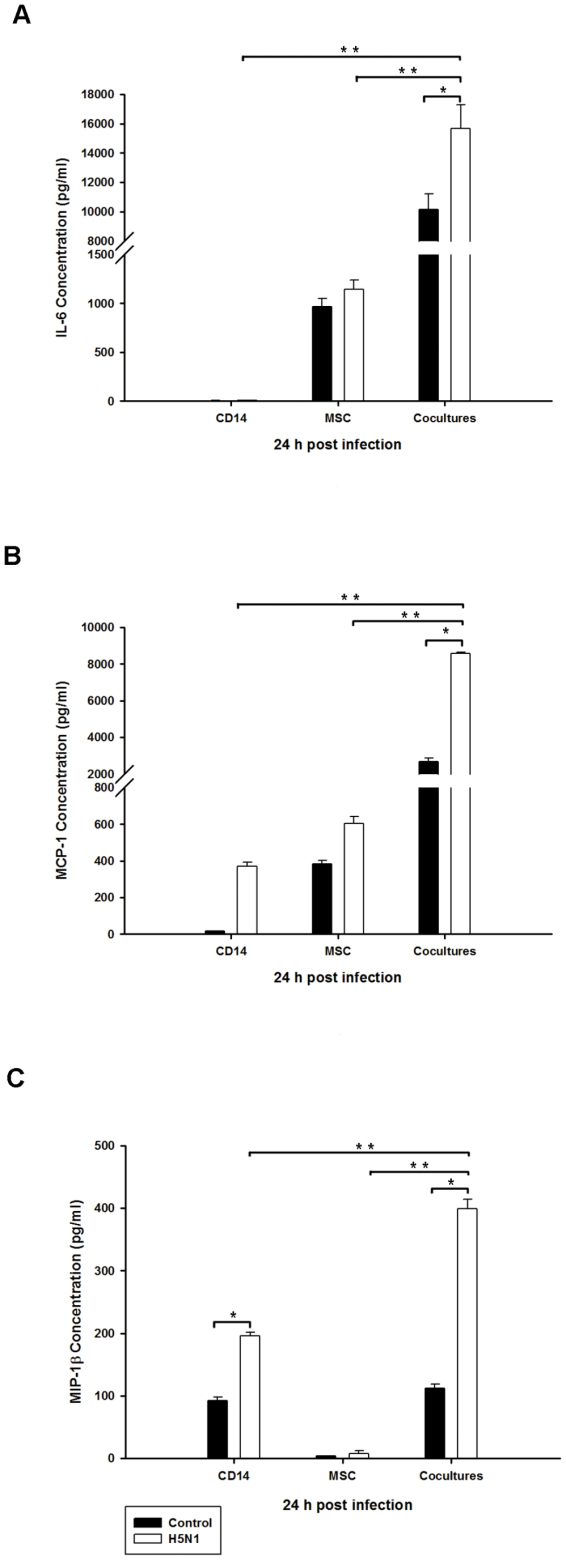
Production of IL-6, MCP-1, and MIP-1β from H5N1-infected cocultures. (**A**) IL-6, (**B**) MCP-1, and (**C**) MIP-1β levels from supernatants of infected CD14^+^ monocytes and MSCs monoculture and 1∶5 ratio of MSCs/CD14^+^ monocytes cocultures at MOI 0.04 were measured by using Bio-plex Cytokine assay. Data was analyzed via Bio-plex 5.0 Software. The two different cell types used in the experiment were derived from different donors. The results represent the means and SD of two independent experiments. Single asterisk indicates statistically significant differences between mock and infected cells with *P* values of <0.05 and double asterisks indicate statistically significant differences between cocultures and monoculture groups with *P* values of <0.05.

Considering MSCs are non-immune cells that possess an immunomodulative activity, further exploring the role of MSCs in H5N1 pathogenesis is crucial. Previous reports demonstrated that MSCs are capable of suppressing the differentiation of monocyte-derived dendritic cells (MoDCs) [15], [17]. It is interesting to investigate the immune dysregulation following H5N1 infection. Therefore, we hypothesized that H5N1 infection could alter MSCs-mediated immune modulation. Monocytes/macrophages secreted mainly inflammatory mediators including IL-1β, IL-6, IL-8, MCP-1, MIP-1β and GM-CSF which are known to be key players in the H5N1-mediated immunopathogenesis [28], [29]. Here, we designed an experiment in which MSCs/monocyte cocultures was infected with H5N1 virus at an MOI of 0.04, and alterations in the cytokine profiles of infected cocultures were compared to those of mock, MSCs monoculture, and monocyte monoculture. At 24 hours post-infection, culture supernatants were collected to determine cytokine production. Cytokines and chemokines were measured by using a Bio-plex Pro Human Cytokine Assay. We found that IL-6 level of H5N1-infected MSCs was comparable with mock, whereas it was barely detectable in infected CD14^+^ monocytes (Figure 5A). The chemokines; MCP-1 and MIP-1β were not up-regulated in H5N1-infected MSCs monoculture, but slightly increased in infected CD14^+^ monocytes (Figure 5B and 5C). IL-6, MCP-1 and MIP-1β were significantly up-regulated in the H5N1-infected MSCs/Monocytes cocultures compared with mock, and both monocultures (Figure 5A–5C). In contrast, some cytokine and chemokines such as IL-1β, IL-8, and GM-CSF from H5N1-infected cocultures were not significantly different from their respective controls (Figure S5). These findings demonstrated that H5N1-infected MSCs/Monocytes cocultures could produce high level of IL-6, MCP-1 and MIP-1β indicating that H5N1 infection has a potential to induce immune dysregulation of MSCs when they were cocultured with monocytes.

## Discussion

Details regarding the pathogenesis of H5N1 are limited. H5N1 infection is not restricted to respiratory organs, and this systemic spread of the virus makes H5N1 unique from other human influenza viruses. Detection of viral RNA in BM upon autopsy and the presence of hematologic abnormalities in severe H5N1 cases increased our interests in H5N1-mediated BM suppression. It is therefore important to investigate viral tropism in BM cells. Previous studies of direct infection of CD34^+^ HSCs and MSCs by other persistent viruses, such as Measles virus (MV) [10] and Cytomegalovirus (CMV) [30], [31] have demonstrated virus-mediated immunosuppression. However, acute viral infection of BM cells remains unclear.

In the present study, we first demonstrated that H5N1 virus could directly infect primary human BM progenitor cells: HSCs and MSCs. Viral infection induced cell death and affected the immune modulation activity of MSCs. Our obtained data could better explain viral pathogenesis in extra-pulmonary organs. We started with isolation of CD34^+^ HSCs and MSCs from the BM and CB of healthy donors. Using different techniques including flow cytometry, quantitative RT-PCR, plaque assay, immunofluorescence and confocal microscopy, we demonstrated that CD34^+^ HSCs and MSCs supported a productive infection of H5N1 virus, although to a lesser degree in CD34^+^ HSCs. Both stem cells were resistant to human influenza virus (H1N1 and H3N2) infection, even though these two cells expressed both α-2,3 SA and α-2,6 SA, receptors for avian and human influenza viruses, respectively. Our findings suggested that H5N1 virus had BM tropism and CD34^+^ HSCs and MSCs could serve as key amplifiers of virus within the BM compartment. The mobilizing property of these two cells could support a spread of virus throughout the body [9], [32]. Our findings are consistent with Gu J., *et al*. [33] and Uiprasertkul M., *et al*. [34] studies that demonstrated H5N1 was able to disseminate and infect extra-pulmonary organs showing a broad tissue tropism of virus. However, there is a limited autopsy data to prove a dissemination of virus in systemic organs.

The susceptibility of each cell type to H5N1 infection is partly dependent on SA receptors since some α-2,3 SA and 2,6-SA expressing cells are unable to support efficient infection and replication of virus [35]. In addition, there might be some unknown intracellular factors or mechanisms that involved with permissibility of CD34^+^ HSCs to H5N1 virus. Previous studies have demonstrated that H5N1 could infect various targets in particular cells of the immune system [21], [36]–[38], it is possible that H5N1 is able to evade the host innate immune response with multiple mechanisms such as NS1-mediated IFN inhibition, the proapoptotic function of PB1-F2 in limiting efficient immune cell-mediated virus clearance *in vivo*, and the receptor switching of avian to human receptors enabling H5N1 to evade the virus-neutralizing effects of mucins containing α-2,3 SA [4], [39]. Further investigations should be performed to understand the mechanism underlying high susceptibility of progenitor cells to H5N1 over H1N1 and H3N2 viruses, although these two viruses are more likely to be laboratory strains than human influenza strains.

Studies have shown that apoptosis plays a major role in H5N1 pathogenesis. Apoptosis was induced upon infection of epithelial cells and lymphocytes *in vitro* [40], [41]. Increased apoptosis in human lymphocytes leading to severe lymphopenia which was caused by either direct infection or over-activation of the cytokine response [41]. In this study, H5N1 virus induced high rates of apoptosis in both MSCs and CD34^+^ HSCs. Apoptosis observed in a majority of infected MSCs was likely due to direct infection, whereas, apoptosis in CD34^+^ HSCs might be induced by paracrine factors as apoptotic signals were observed in uninfected CD34^+^ HSCs population (Figure 4A). Additional experiments to confirm unusual apoptosis in CD34^+^ HSCs will be further investigated. Since CD34^+^ HSCs and MSCs are primitive progenitor cells that contain self-renewal ability and generate descendant cells during hematopoiesis and mesengenic processes, the reduction of precursor cells may affect these two processes which eventually decrease the number of differentiated cells in all lineages. The disruption of hematopoiesis was observed in depleted stromal and CD34^+^ populations by MV infection, which significantly impaired repopulation of lymphoid precursors following MV-induced lymphopenia [10]. Our findings supported abnormal hematologic findings including low peripheral blood counts and cytopenia which are prominent clinical features in patients with severe H5N1 infection. In addition, substantial loss of MSCs would affect the immunosuppressive activity, leading to hyperactivation of the immune response.

The pathogenesis of the H5N1 virus was characterized by broad tissue tropism, systemic replication and hypercytokinemia [42], [43]. H5N1-induced pro-inflammatory cytokines were generated by various immune cells as they coordinate an attack on invading pathogens. Under normal conditions, anti-inflammatory cytokines are immunoregulatory molecules that regulate pro-inflammatory responses. On the other hand, under pathological conditions, disruption of the balance of the anti-inflammatory response to the pro-inflammatory response may occur [44]. MSCs have potent immunoregulatory actions that make them attractive targets for reducing the inflammation and injury [18], [45]. MSCs have been shown to interact with CD14^+^ monocytes and block monocytes differentiation to dendritic cells (DCs) which IL-6 is partially involved in this action [15], [17], [46], [47]. In addition, MSCs can modulate cytokine production by dendritic and T cell subsets [15]. The fact that interaction of MSCs and monocytes leads to immunopathology and dysregulated MSCs-mediated immunomodulation during infection is controversial. Interestingly, our result demonstrated that IL-6, MCP-1, and MIP-1β from MSCs/CD14^+^ monocytes cocultures were up-regulated, whereas this effect was not seen in MSCs or monocytes monoculture. Elevated IL-6 may enhance inflammation as shown in previous studies [43], [48], [49]. It is possible that H5N1 may subvert MSCs-mediated immune modulation by skewing immune modulator towards enhancer as evidenced by the significantly increased levels of IL-6 in infected cocultures. Higher levels of IL-6 are likely to promote inhibition of DC maturation contributing to a reduction in the number of antigen presenting cells which thus leads to slow virus clearance. Previously published data reported that H5N1 was a potent inducer of MCP-1 and MIP-1β which were responsible for the recruitment of immune cells into the infected site [49], [50]. Therefore, high levels of MCP-1 and MIP-1β secreted by infected cocultures may amplify the inflammatory response by attracting many of immune cells into the BM milieu [51] indicating that MSCs might involve indirectly with H5N1-induced inflammation since they are more active responding to H5N1 infection when they are cocultured with monocytes.

Hyperactivation of cytokines, including TNF-α, soluble IL-2 receptor, IL-1 and IL-6 may play a role in the pathogenesis of reactive hemophagocytosis syndrome (RHS) [52]. High levels of IL-6, MCP-1, and MIP-1β production in H5N1-infected cocultures are likely to induce RHS in BM. RHS is characterized by activated macrophages which consequently engulf neighboring cells including hematopoietic cells [53]. This can contribute to markedly hypocellular BM. To *et al*., previously documented that both hypocellular conditions and RHS in BM were the most prominent pathologic features observed in fatal H5N1 cases [54].

Besides BM tissue, MSCs have been identified in many other organs with similar functions including the lung [32], [55]. They are important for the maintenance of lung homeostasis and repair following lung injury [32]. As lung is the primary site of influenza infection, it is possible that lung-resident MSCs may get infected by H5N1 virus resulting in disruption of renewal and differentiation processes. Also, MSCs-mediated immunomodulatory function may be dysregulated as hypothesized contributing to severe injury in the lung. Although there has been no data in humans, influenza virus-infected lung MSCs were investigated in the lungs of both chicken and swine [23], [56].

In summary, we demonstrated that H5N1 virus more efficiently infected and replicated in CD34^+^ HSCs and MSCs compared to human influenza viruses *in vitro*. Apoptosis was a direct result of infection in MSCs, whereas, it might be induced by paracrine factors in CD34^+^ HSCs. In addition, H5N1 infection could interfere with MSC-mediated immune modulation by shifting immune modulator production towards enhancers, which, in turn, amplifies the cytokine response by the influx of circulating monocytes and macrophages. Our explorations provide an insight into H5N1 pathogenesis in terms of aggressive systemic infection and hematologic abnormalities in patients with severe H5N1 infection.

## Supporting Information

Figure S1 Susceptibility of CB-derived CD34^+^ cells to different H5N1 strains. Infection is determined by using a specific marker (Nucleoprotein), and detected by flow cytometry. Cells were infected by different strains of avian and human influenza viruses at an MOI of 10 for 24 h. The results were obtained individually from three different experiments and are presented as means plus standard errors. *P<0.05 indicates statistically significant differences between H5N1 infection and human influenza viruses infection (H1N1 and H3N2).
(TIF)

Figure S2 Viral production of H5N1-infected MSCs at various doses and incubation times. Avian influenza H5N1 could infect and replicate in MSC. Data are plaque-forming units in the supernatant of MSCs infected with H5N1 at various MOI for 1 h, washed, and incubated for indicated time points.
(TIF)

Figure S3 High susceptibility of MSCs was restricted only to HPAI H5N1. MSCs were infected with rgPR8-H5, rgPR8-H5monobasic, and rg-PR8 which represent HPAI H5N1, LPAI H5N1, and H1N1 viruses, respectively at MOI 1 for 24 hours. LPAI H5N1 is the reverse genetics (rg) virus bearing monobasic amino acids at HA cleavage site as described in materials and methods section. Percentages of infection were determined by flow cytometry. The results represent the means and SD of two independent donors. *P<0.05 indicate statistically significant differences of rgPR8-H5 compared with rgPR8-H5monobasic, and rg-PR8 viruses.
(TIF)

Figure S4 Percentage of apoptotic cells induced by H5N1 infection. (A) CD34^+^ HSCs were infected with H5N1 virus and (B) MSCs were infected with both avian and human influenza viruses at an MOI of 10. After 18 h, cells were fixed, permeabilized and multiple stained with TUNEL, NP and DAPI and determined by confocal microscopy as described in Figure 4. The percentages of positive signal were obtained by counting positive cells from five random views. Data are given as mean ± SD of two independent experiments. *P<0.05 indicates statistically significant differences between the percentages of TUNEL+ and Merge (NP+TUNEL+) of H5N1-infected CD34^+^. **P<0.05 indicates the significance of the number of H5N1-induced apoptosis compared with human influenza viruses in MSCs.
(TIF)

Figure S5 H5N1 did not induce IL-1β, IL-8, and GM-CSF in cocultures. (A) IL-1β, (B) IL-8, and (C) GM-CSF levels were measured using a Bio-plex Cytokine assay. The two different cell types in this experiment were derived from different donors. Data shown are mean±SD of two independent experiments. Single asterisk indicates statistically significant differences between mock and infected cells with *P* values of <0.05 and double asterisks indicate statistically significant differences between cocultures and monoculture groups with *P* values of <0.05. n.s. means no significance.
(TIF)
