# Supplementary figures and images for: Tropism of Avian Influenza A (H5N1) Virus to Mesenchymal Stem Cells and CD34+ Hematopoietic Stem Cells
Source: PLoS One. 2013 Dec 10;8(12):e81805. doi: 10.1371/journal.pone.0081805 (PMC3858287; doi:10.1371/journal.pone.0081805)

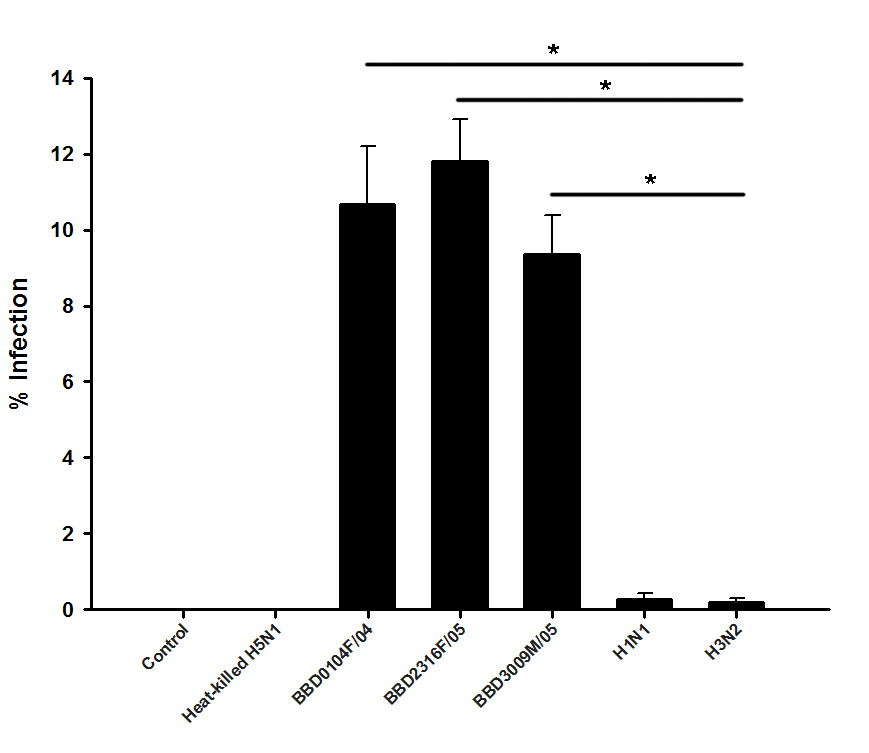

Supplement: Figure S1 — Susceptibility of CB-derived CD34+ cells to different H5N1 strains. Infection is determined by using a specific marker (Nucleoprotein), and detected by flow cytometry. Cells were infected by different strains of avian and human influenza viruses at an MOI of 10 for 24 h. The results were obtained individually from three different experiments and are presented as means plus standard errors. *P<0.05 indicates statistically significant differences between H5N1 infection and human influenza viruses infection (H1N1 and H3N2). (TIF) [file pone.0081805.s001.tif]

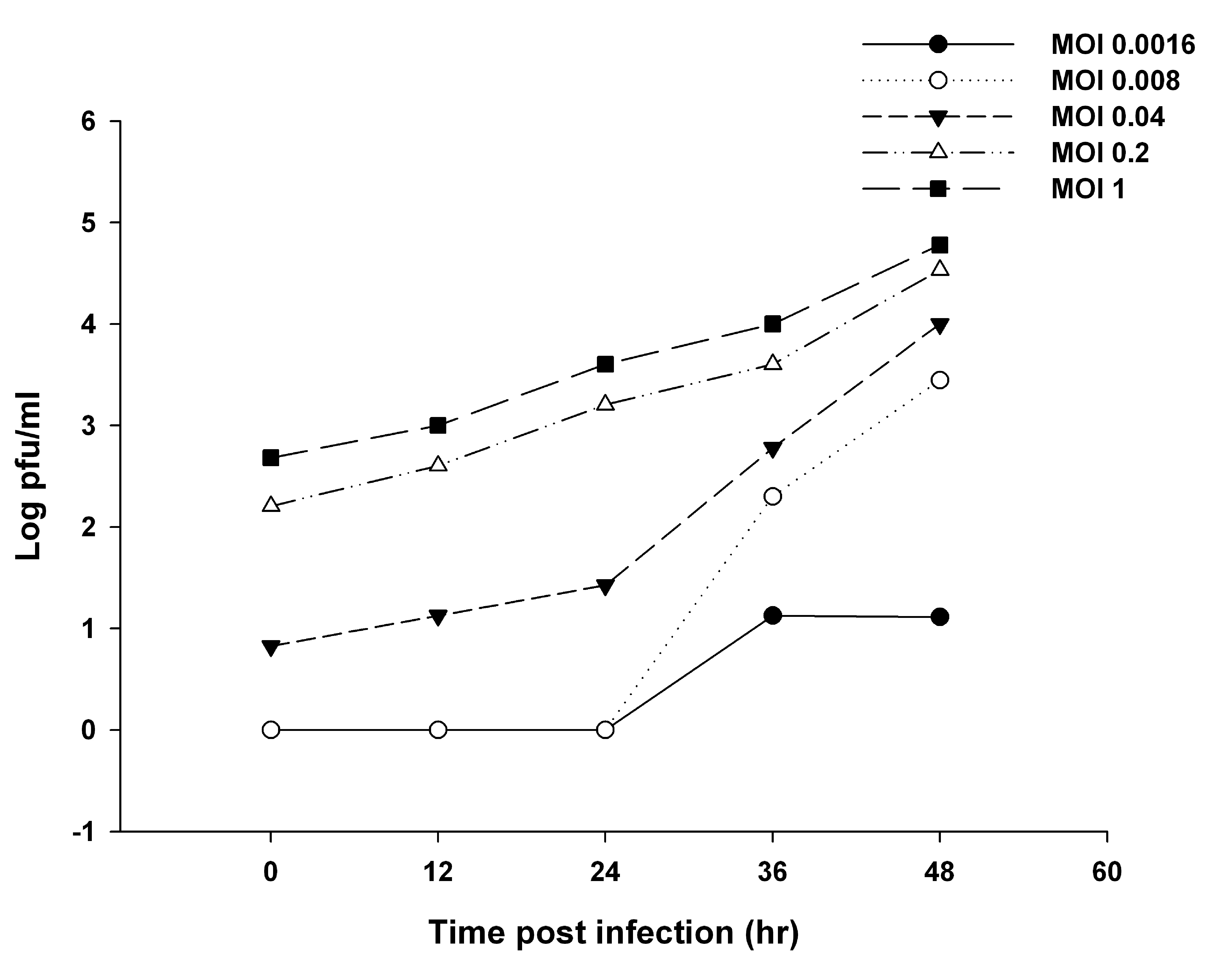

Supplement: Figure S2 — Viral production of H5N1-infected MSCs at various doses and incubation times. Avian influenza H5N1 could infect and replicate in MSC. Data are plaque-forming units in the supernatant of MSCs infected with H5N1 at various MOI for 1 h, washed, and incubated for indicated time points. (TIF) [file pone.0081805.s002.tif]

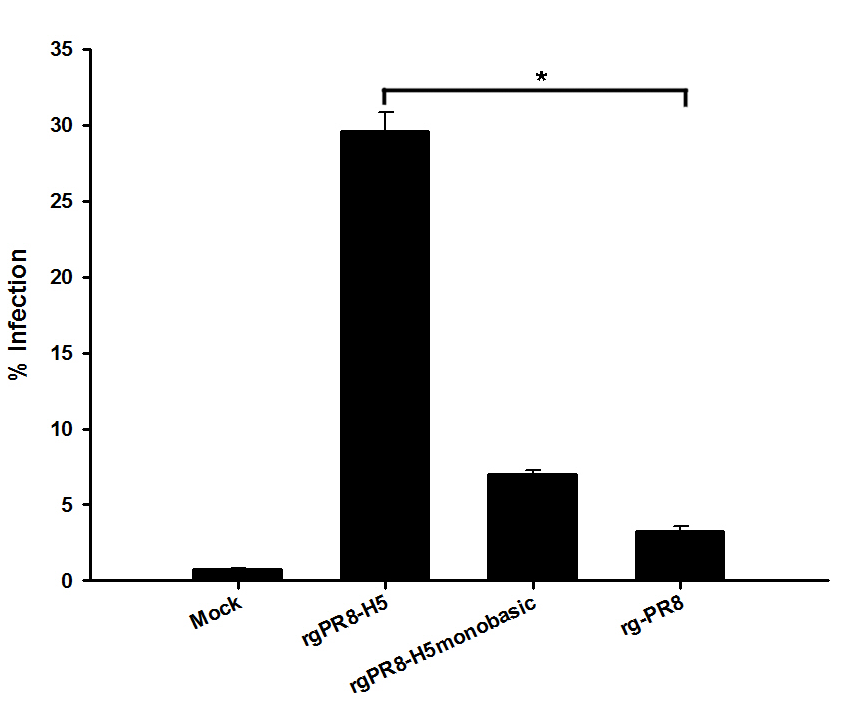

Supplement: Figure S3 — High susceptibility of MSCs was restricted only to HPAI H5N1. MSCs were infected with rgPR8-H5, rgPR8-H5monobasic, and rg-PR8 which represent HPAI H5N1, LPAI H5N1, and H1N1 viruses, respectively at MOI 1 for 24 hours. LPAI H5N1 is the reverse genetics (rg) virus bearing monobasic amino acids at HA cleavage site as described in materials and methods section. Percentages of infection were determined by flow cytometry. The results represent the means and SD of two independent donors. *P<0.05 indicate statistically significant differences of rgPR8-H5 compared with rgPR8-H5monobasic, and rg-PR8 viruses. (TIF) [file pone.0081805.s003.tif]

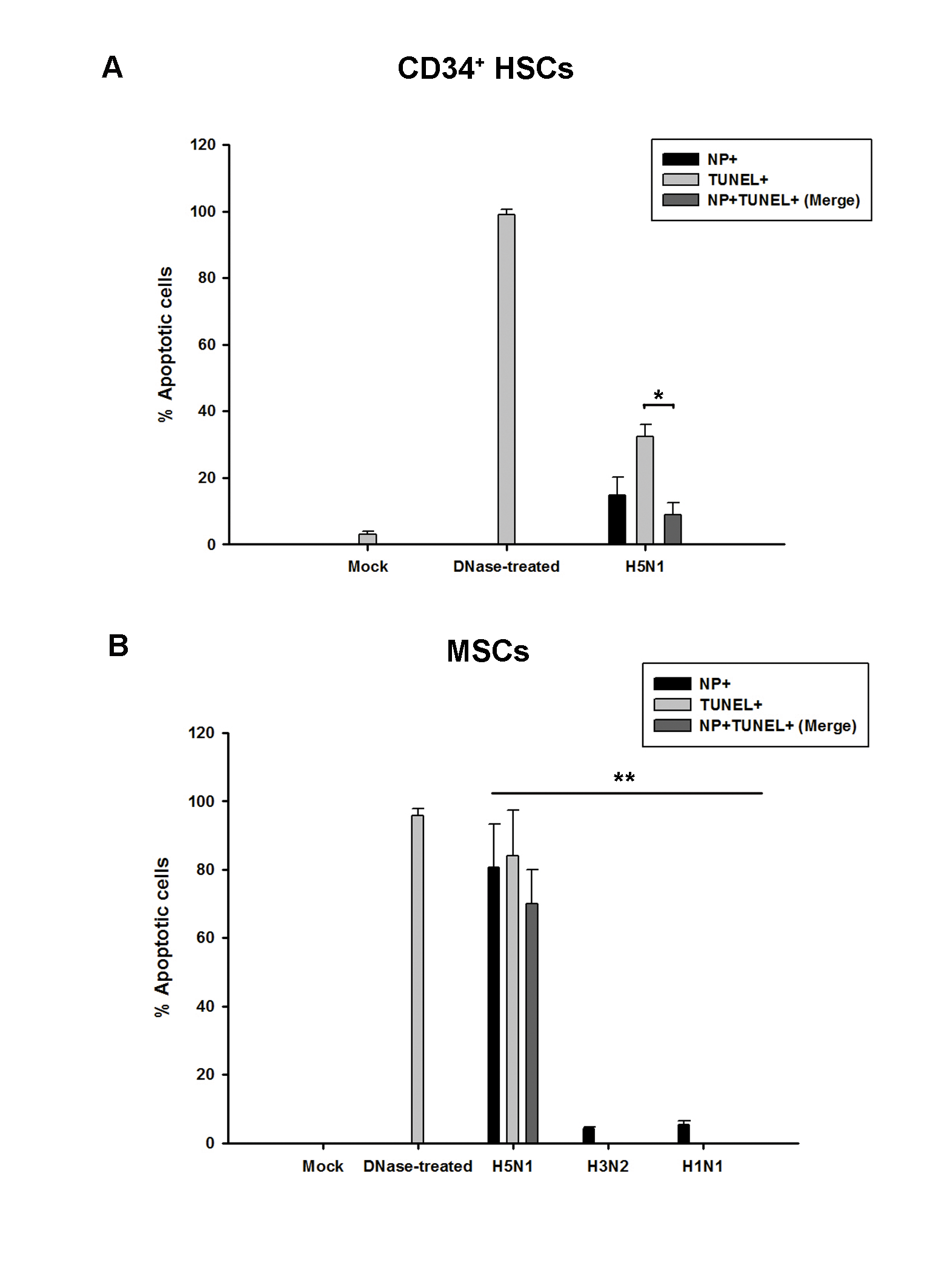

Supplement: Figure S4 — Percentage of apoptotic cells induced by H5N1 infection. (A) CD34+ HSCs were infected with H5N1 virus and (B) MSCs were infected with both avian and human influenza viruses at an MOI of 10. After 18 h, cells were fixed, permeabilized and multiple stained with TUNEL, NP and DAPI and determined by confocal microscopy as described in Figure 4. The percentages of positive signal were obtained by counting positive cells from five random views. Data are given as mean ± SD of two independent experiments. *P<0.05 indicates statistically significant differences between the percentages of TUNEL+ and Merge (NP+TUNEL+) of H5N1-infected CD34+. **P<0.05 indicates the significance of the number of H5N1-induced apoptosis compared with human influenza viruses in MSCs. (TIF) [file pone.0081805.s004.tif]

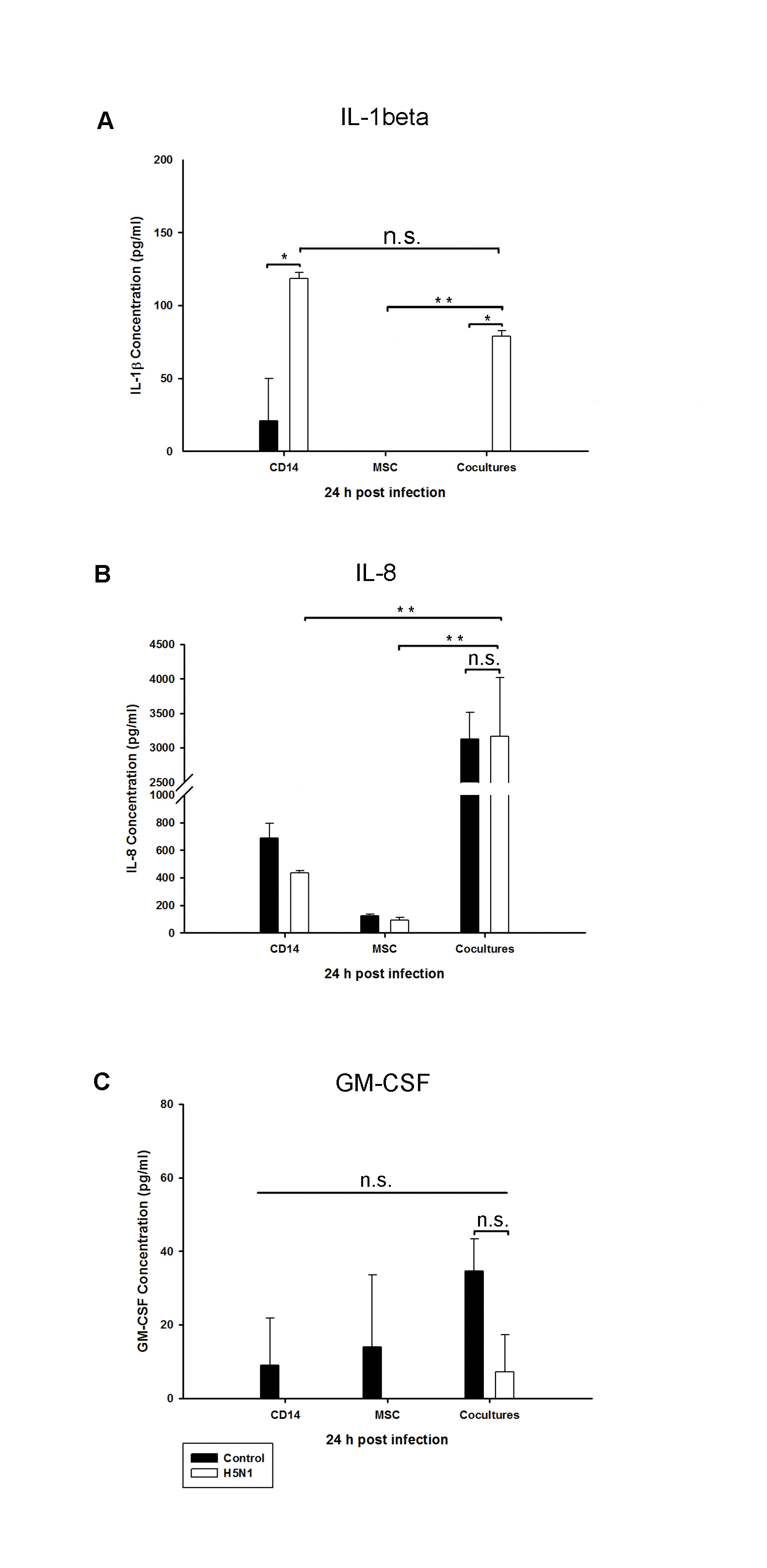

Supplement: Figure S5 — H5N1 did not induce IL-1β, IL-8, and GM-CSF in cocultures. (A) IL-1β, (B) IL-8, and (C) GM-CSF levels were measured using a Bio-plex Cytokine assay. The two different cell types in this experiment were derived from different donors. Data shown are mean±SD of two independent experiments. Single asterisk indicates statistically significant differences between mock and infected cells with P values of <0.05 and double asterisks indicate statistically significant differences between cocultures and monoculture groups with P values of <0.05. n.s. means no significance. (TIF) [file pone.0081805.s005.tif]
